# A short guide to topological terms in the effective theories of condensed matter

**DOI:** 10.1088/1468-6996/16/1/014404

**Published:** 2015-02-18

**Authors:** Akihiro Tanaka, Shintaro Takayoshi

**Affiliations:** Computational Materials Science Unit, National Institute for Materials Science, Namiki 1-1, Tsukuba, Ibaraki 305-0044, Japan

**Keywords:** topological terms, effective action, quantum magnetism, superfluids, symmetry-protected topological phases

## Abstract

This article is meant as a gentle introduction to the topological terms that often play a decisive role in effective theories describing topological quantum effects in condensed matter systems. We first take up several prominent examples, mainly from the area of quantum magnetism and superfluids/superconductors. We then briefly discuss how these ideas are now finding incarnations in the studies of symmetry-protected topological phases, which are in a sense a generalization of the concept of topological insulators to a wider range of materials, including magnets and cold atoms.

## 1. Introduction

Topological quantum effects, whose significance to material functions is gaining wide recognition with the discovery of the topological insulator, have actually been (along with electron correlation effects) one of the core pillars of contemporary condensed matter physics since the 1980s. It thus seems appropriate to try to achieve an understanding of problems where topology plays a governing role more or less as a whole: such an attempt will hopefully help fit the ongoing topics taken up in this issue into a larger physical frame, while the older problems can be revisited in light of the latest developments.

The purpose of this article is to take a microscopic step toward that goal by taking a cursory glance at the role played by topological terms, which typically show up in the low-energy action of systems where topological quantum effects take place. The emphasis is on pedagogy, and no attempt is made on completeness or rigor. Our modest objective is to warm the interested nonspecialist reader to some of the basic features of topological terms through simple examples, preparing her/him to be exposed to the vast literature on this rapidly evolving subject.

There are monographs which cover similar grounds but from a broader perspective [1–4], to which we refer the reader for an in-depth exposition. Altland and Simons [4] in particular offer a readable discussion on the defining mathematical structures of topological terms, which will not be detailed here.

## 2. The two uses of path integrals in condensed matter

The format we employ below is that of path integrals, which are used in a good many theoretical works on this subject. Aside from being the natural language for modern quantum field theory, which is where topological terms originate from, it proves to be particularly convenient when it comes to extracting the low-energy properties of a many-body system, especially those of a topological nature.

There are mainly two ways in which path integrals come into play in our discussion. One is in evaluating the probability amplitude of a quantum mechanical process, i.e. a complex number whose squared modulus yields the probability. Suppose we are studying a field *ϕ* (generally a function of time *t* and position 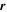) which, in our examples below, is typically an order parameter for some condensed matter (such as a superconductor or a ferromagnet). If we are interested in the probability amplitude associated with the realization of a certain spatial configuration 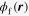, we can formally write it down as the path integral (for most of the following we will set 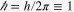)1
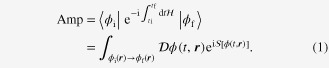
The first line is the textbook expression for this quantity which involves the time evolution kernel 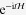, where 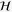 is the Hamiltonian of the system (suitable generalizations are to be made when 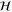 at different times do not commute). In the path integral formula in the second line, the integration stands for a summation over all possible ways in which *ϕ*, which at an initial time took the configuration 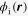, evolves at the final time into the configuration 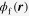. Each version of these evolution histories is called a path, and is weighted by the Feynman weight 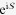, where 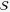 is the action. In what follows it will often prove to be convenient to convert from real time (*t*) to the imaginary time (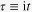) formalism. Although mathematically this can be a highly nontrivial procedure, the formal prescription for this conversion is actually rather simple and consists of two steps. First we make the substitution 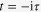 in equation (1). Following this, we *define* the imaginary time action 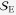 using the original real time action 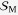 action (the subscripts each stand for Euclidean and Minkowskian spacetime) through the correspondence 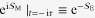. Although we will suppress the subscripts E and M below for the sake of notational brevity, we will specify which of the two frameworks we are working with. It can be shown [5] that by continuing to imaginary time and then evaluating the above path integral expression for the amplitude, which now takes the form 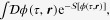 we can effectively project our theory onto the ground state. We will later take advantage of this fact to evaluate the ground state wavefunctional 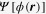, i.e. the probability amplitude for the configuration 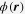 to be realized in the ground state.

The second usage is to gain information on the partition function 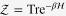 which arises in quantum statistical mechanics. To convert to a path integral representation, one breaks up the Boltzmann weight into a product of many exponential operators, 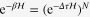, where 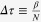. Since each 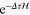 can be viewed as the generator of time evolution over a interval 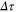 in imaginary time, the partition function can be written, on taking the limit 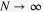, as the imaginary time path integral2
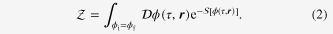
Here we are summing over paths in which the field 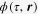 obeys a periodic boundary condition in the imaginary-time direction, which reflects the trace operation that is involved in calculating the partition function.

The definition of a topological term 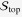 varies considerably among authors. For instance, one can adopt the view that they are the actions which are metric-independent, i.e. do not change form with the topology of spacetime [4]. Here we use the terminology in its broadest sense, and associate it with actions which have the following two features. (1) It is the portion of the action which arises *in addition to* the kinetic action coming directly from the Hamiltonian 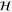, i.e. the total action should generally have the form 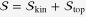, where (in imaginary time) 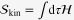. (2) The term 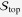, when using the imaginary time framework is *purely imaginary* and hence contributes a phase factor to the Boltzmann weight 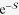. (For a simple single particle quantum mechanics example on this aspect see Auerbach [1] or Altland-Simons [4].) The first feature implies that the topological term usually has no apparent classical counterpart, while the second feature is suggestive of nontrivial quantum interference effects which may arise when an action contains a topological term. Thus it is often an indication of interesting physics. It is also often the case, as justifies its name, that 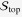 bears a clear topological significance, such as a proportionality to a topological winding number. We will encounter a number of such examples in the following.

Deriving, or establishing the existence of a topological term in the low energy effective theory of a system starting from a given Hamiltonian, can be a subtle and highly technical issue. (For example, whether or not a topological term known as the Hopf term should arise in the effective action of 2D Heisenberg antiferromagnets was briefly controversial [2].) Our plan therefore will be to examine, without going into the derivation itself, illustrative examples where topological terms are known to exist, and explore how their presence will alter the low-energy physics.

## 3. Quantum interference

Suppose that the total effective action of the system in consideration consists of a kinetic term plus a topological term, i.e. 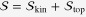. As mentioned in the previous section, the probability amplitude associated with the transition 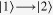 is, in path integral language, 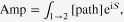 where the integration symbol simply means that a summation over all paths for which the system is in state 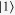 at initial time and state 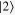 at final time be taken. Important consequences can arise if this summation is dominated by paths which cost the system essentially the same kinetic energy, but differ in the value of the topological term. The amplitude then factorizes into the form3
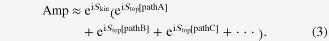
Clearly the topological term can lead to a quantum interference effect: in particular, if the factors 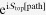 sum up to zero (i.e. a destructive interference), the transition in question is forbidden, even though the transition amplitude for each path 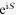 is nonzero. (This scenario applies for both the real time and the imaginary time path integral.) We now take up the following several examples where this happens.

### 3.1. Single spin: nanomagnet

**Figure 1.**
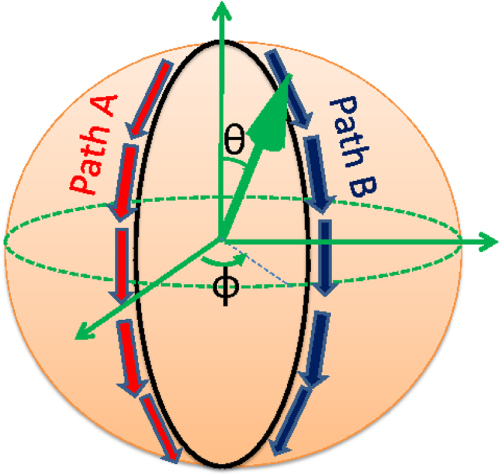
The two low-energy paths relevant to the evaluation of the transition amplitude from the up-spin state to the down-spin state. The difference of the Berry phase for the two paths is well defined and equals *S* multiplied by 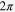, i.e. the surface area corresponding to half of the unit sphere.

Consider a spin moment with spin quantum number *S*. Suppose that there is a dominant easy-plane and a sub-dominant easy-plane anisotropy; we take for concreteness the Hamiltonian 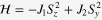, with 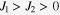. If we denote the direction of the spin vector by the unit vector 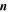 (the direction is only meaningful in a semi-classical sense since the spin components are non-commuting entities), the action is given by4
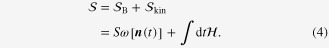
The topological term 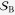 is related to the Berry phase which is induced by the evolution of the spin orientation. If the motion of 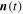 is such that its orientation coincides at the beginning and the end of the time interval under consideration, this term can be expressed as in the second line, where 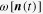 is the solid angle, i.e. the area which the trajectory of 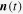 traces out on the surface of the unit sphere. In terms of the spherical coordinate 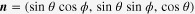, this is explicitly written as5
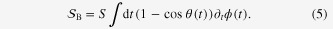
Let us now consider the probability amplitude associated with the transition of 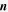 from the north to the south pole, i.e. between the two lowest energy states [6, 7]. The path integral for this transition is dominated (see figure 1) by the two low energy paths connecting the poles, which are located along the longitudes 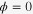 (path A) and 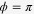 (path B). Though each path alone does not trace out a closed curve, their *difference* does. Noting that the two paths correspond to the same kinetic energy, we expect the transition amplitude to take the form6
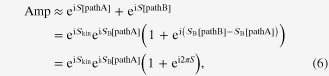
where, in the third line, we have used the fact that the surface area corresponding to half of the sphere is 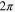. (A detailed evaluation can be found in the original references [6, 7].) Thus if *S* is half of an odd integer (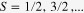), the probability amplitude vanishes (destructive interference), while if *S* is integer-valued (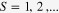), the amplitude is enhanced (constructive interference). This can have experimental consequences, for example, for switching effects in single-molecule nanomagnets such as Mn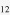-acetate and Fe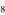. The two interference patterns discussed above can manifest themselves as unavoided or avoided level crossings between the up-spin and down-spin levels (the former case corresponds to the suppression of hybridization) as a function of an external magnetic field applied along the *z*-axis. (It has been pointed out however that realistic situations tend to obscure this effect [8].) It is also interesting that essentially the same mechanism has been proposed to control the macroscopic tunneling of magnetic flux in a fabricated superconducting island (it is possible to map the low-energy sector onto an effective spin system), which may find applications to quantum information processes [9].

### 3.2. Superconductor and superfluid

We turn to the effective low-energy action of a superfluid or a superconductor; we will focus on the former as most of what follows can be generalized to the latter by simply coupling the system to an electromagnetic field. Assuming that the amplitude of the condensate’s order parameter is well-developed, the low energy physics can be described solely in terms of the phase degree of freedom *ϕ*, and the effective action typically takes the form [11] (with applications to the partition function in mind, we employ the imaginary time formalism)7
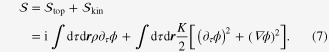



**Figure 2.**
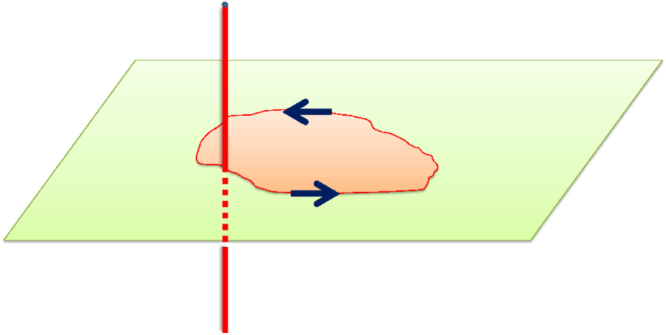
The trajectory of a vortex executing a round excursion through a superfluid (of superconductor) thin film. The Berry phase associated with this process, which is recorded by the topological term, is proportional to the number of particles within the area bounded by the closed curve. In an analogy with the Aharonov–Bohm effect, this indicates that the vortex behaves like a charged particle in an external magnetic field.

The second term on the right-hand side can be derived from a standard Ginzburg–Landau type action using a phase-only approximation. (The coefficient *K*, which describes the rigidity against phase fluctuations, is proportional to the square of the amplitude of the order-parameter. We also note that we have set for simplicity a coefficient with the dimension of a velocity to unity.) The first term is the topological or Berry phase term, and resembles the 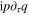-term which appears in the (imaginary-time) Lagrangian of a single particle whose dynamics is described in terms of a pair of canonically conjugate variables *q* (position) and *p* (momentum). Recalling that the canonical conjugate of the phase *ϕ* is the particle density of the superfluid condensate, we interpret the coefficient *ρ* as the superfluid density (or more precisely, the offset value that this term imposes on this physical quantity). To see how this term influences the physics of the superfluid, we will study the motion of a vortex moving about in the system; for simplicity we consider a 2D system, i.e. a superfluid thin film, with a constant value of *ρ*. Furthermore, we assume that the vortex returns after an excursion to its initial position (see figure 2), as is required from the periodicity in the imaginary time direction. It is easy to check that the action 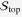 contributes the quantity 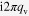 each time the vortex goes around a (bosonic) particle in a superfluid condensate (or in superconductor language, a Cooper pair), where 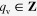 is the vorticity. Hence, if the total number of bosons which has been encircled by the vortex is 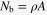, where *A* is the area bounded by the trajectory (note that in the 2D case *ρ* is the number of condensate particles per area, and 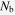 generally is *not* an integer), the net outcome from the topological term becomes 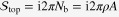. In other words, it contributes to the Boltzmann weight 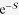 entering the path integral a phase factor of8
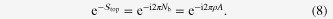
This may be viewed as an Aharonov–Bohm-like effect; vortices see the surrounding condensate particles as a sort of magnetic field (with an intensity proportional to *ρ*), as is clear from the phase accumulation that occurs when the vortex performs a round trip.

**Figure 3.**
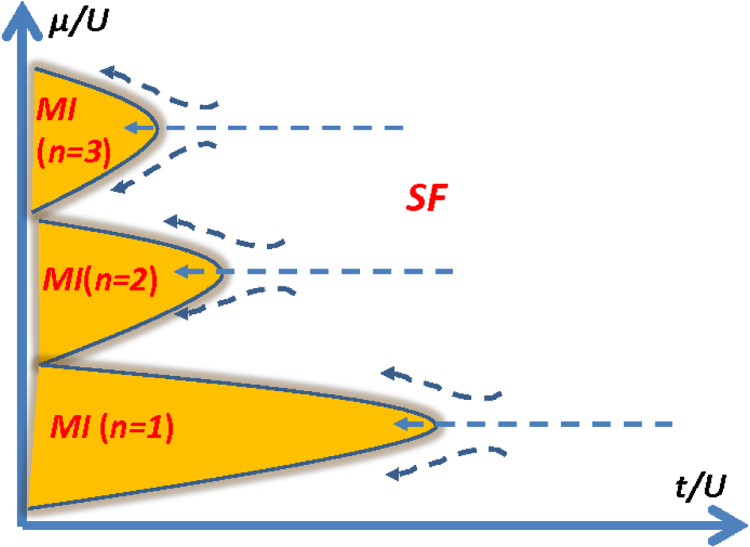
A schematic phase diagram of the boson Hubbard model. SF and MI stand for the superfluid and Mott insulator phases. The dotted arrows denote the constant *ρ* contours. When the boson filling factor is an integer, i.e. 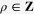, the system can undergo a SF to MI transition upon reducing the parameter 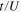. For generic filling factors, the superfluid phase persists. This can be understood in terms of a destructive interference among vortex excitations due to the presence of the topological term.

The phase factor of equation (8) can lead to important consequences. Generally, when a condensation of vortex excitations in a superfluid condensate (point vortices in 2D, vortex loops in 3D, spacetime vortices, or phase-slip events in 1D) occurs, it will destroy the superfluidity, and the system is expected to enter a new phase. While we are familiar with these types of phase transitions that occur at finite temperatures, they can also happen at zero temperature with the variation of some control parameters. Quantum phase transitions of this variety can be studied in detail using the boson Hubbard model, which has recently attracted much attention due to its direct relevance to the physics of cold atoms. This model has the following lattice Hamiltonian [12], 9
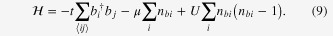
The summation in the first term is to be taken with respect to nearest neighbor sites, *b*_*i*_ (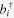) is a boson annihilation (creation) operator, 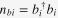 is the boson number operator, and *μ* and *U* are each the chemical potential and the onsite Coulomb repulsion. Advances in cold atom experiments now enable researchers to access a wide region of the phase diagram of this model, which is depicted in figure 3. When 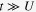, the system is in a superfluid phase, for which the effective action of equation (7) gives a suitable description. When 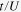 is reduced, the tendency to form a superfluid weakens, and one naively expects to encounter a phase transition into a Mott insulator phase. However, as already noted, this transition proceeds by the condensation of vortices, which are each accompanied by the Berry phase factors of the form written in equation (8). For generic (irrational) values of *ρ*, which translate in the lattice model to generic boson filling factors, these will enter into the path integral as random phase factors. Although further analysis is necessary to work out the details [12], it is then reasonable to deduce that configurations containing vortices will cancel out and effectively drop out from the partition function altogether, meaning that the system cannot undergo a transition into the insulating phase. An exception occurs however when 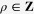, i.e. at integer boson filling, since for this case the phase factor of equation (8) is unity. This expectation can be confirmed by a study of the boson Hubbard model. At integral filling, a transition into the Mott insulator phase occurs at the tip of the Mott insulator phase lobe, while for generic filling factors, the constant-*ρ* contour avoids entering the insulator phase and escapes into the region between the lobes. (The case where the filling factor is a nonintegral rational number requires a careful treatment; the possibility that vortices with some higher vorticity will condense, giving rise to an exotic phase, has to be taken into account and is beyond the scope of the present argument.) Although we have focused in the above on a 2D system, it is straightforward to show that similar reasonings apply to systems of other dimensionalities.

### 3.3. Magnetization plateau

**Figure 4.**
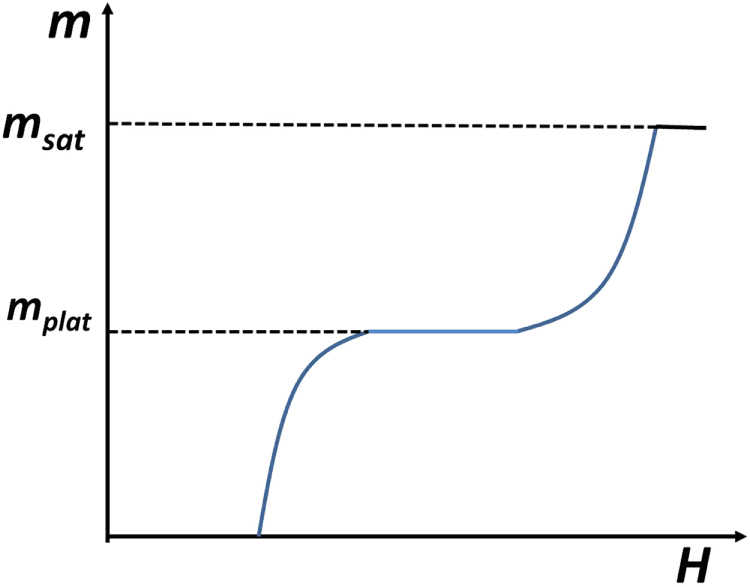
Schematic picture of a magnetization curve (magnetization per site *m* as a function of applied magnetic field *H*) featuring a plateau region at 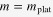. The saturation value of *m* is denoted as *m*_sat_. (Adapted from Kim and Tanaka [10].)

A closely related phenomenon, also driven by the phase interference between vortex Berry phases, occurs in antiferromagnets in an external magnetic field. When one probes the magnetization per site *m* as a function of the external field *H*, there often appear plateau regions in the magnetization curve (see figure 4), contrary to classical analysis which predicts a monotonic increase. The crucial question is when and how the plateau forms.

This can be understood in the following way (though the plateau can occur for general spatial dimensions, we continue to discuss the 2D case). Since the spin component parallel to the magnetic field (assume this to be in the z-direction), i.e. the magnetization, is already optimized by the field, it is not free to participate in the low-energy spin fluctuations (in other words, the fluctuation in the field direction is energetically costly). The low-energy effective theory therefore should consist of the dynamics that take place within the xy-plane, which can be represented by an angular field *ϕ*, as in the superfluid action discussed above. The action [13] in fact turns out to have the same form as equation (7), but with *ρ* substituted by 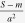, where *S* is the spin quantum number, *m* the magnetization per site, and *a* the lattice constant. Therefore, in analogy with equation (8) a round excursion of a vortex enclosing an area *A* which contains 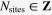 sites will contribute a phase factor of10
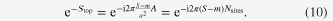
As before, for generic values of 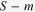 these Berry phase factors will suppress vortex condensation. Condensation may occur, if energetically favorable, when 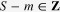, which will disorder the ground state and create a finite energy gap between the ground state and the excited states. This gapful state is none other than the magnetization plateau.

### 3.4. Haldane gap

Perhaps the most well known application of the interference effect of the Berry phase or topological terms to condensed matter systems is the pioneering work by Haldane [14] on antiferromagnetic Heisenberg spin chains described by the Hamiltonian11
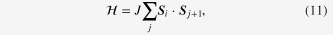
where 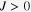. The order parameter for the problem is chosen to be the unit vector 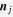 which specifies the orientation of the staggered spin moment, i.e. 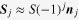.

**Figure 5.**
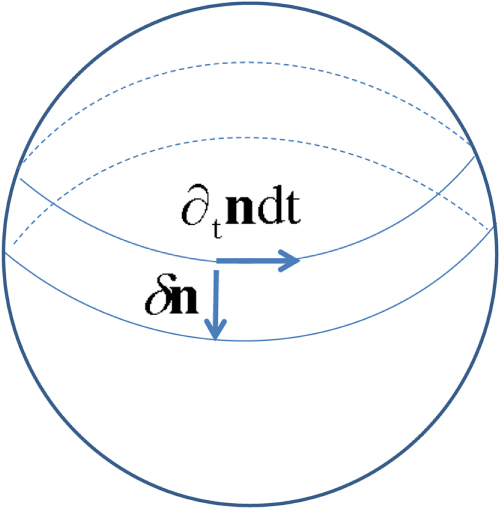
Trajectories corresponding to two slightly varying (imaginary) time evolutions of the unit vector 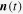. The difference in the solid angle that is traced out amounts to 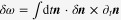.

The kinetic term of the effective action should describe the tendency for nearby 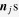 to align, and is found to take the form12
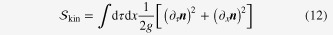
in the continuum approximation, where *g* is a coupling constant which depends on the details of the starting lattice Hamiltonian (we have again set an additional coefficient with the dimension of velocity to unity for simplicity). In order to derive the topological term of the effective action, we begin by observing that the spin Berry phase term 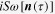 for a single spin moment is odd with respect to spin inversion, i.e. 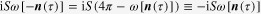. The first equality follows by noting that the solid angle is an oriented surface area, whereas the second equality points to the fact that since this term always appears in the form of a phase factor, the portion 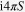 is irrelevant (due to 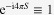). Thus the contributions from the Berry phase term for each spin moment in the antiferromagnet add up into13
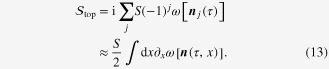
On taking the continuum limit in the second line, we have converted differences into derivatives, and further used the fact that the derivatives are contributed by every other link on the chain (resulting in the factor of 1/2). To proceed, we seek the help of figure 5, and obtain the final form of the topological term (which is often referred to in the literature of a *θ* term),14
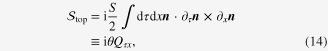
where 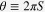, and15
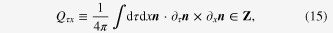
is an integer-valued winding number which counts the number of times 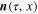 wraps around the sphere as one probes through the entire (Euclidean) spacetime. The partition function therefore reads16
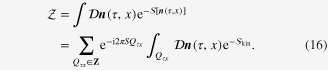
In the second expression we have sorted the configurations entering the path integral according to the value of its winding number 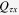. Equation (16) suggests that the system behaves in a qualitatively different way for integer *S* (for which 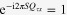) and half odd integer *S* (where 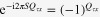). For the latter case, the sign-alternating factors tend to lead to a destructive interference between configurations with nonzero 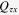 (such spacetime configurations are often called instantons). Since instantons will apparently cause a strong disruption to the antiferromagnet order, we thus expect that antiferromagnetic spin chains with half-odd integer spin, for which case the instanton events are suppressed, should exhibit a stronger degree of spin ordering than those with integer spins, where instantons are *not* suppressed. It is by now well established that the former are indeed critically ordered (i.e. exhibit a power-law decaying spin-spin correlation), while the latter are strongly disordered (with exponentially decaying correlations).

## 4. Quantum dynamics of topological objects

An important aspect of topological terms is that they give rise to unconventional dynamics: since they often describe some variant of the Aharonov–Bohm effect, it is natural to expect that the behavior of the degree of freedom in motion under the influence of the fictitious magnetic field will differ from that of a free particle. This, for instance, lies at the heart of the anomalous Hall effect which is observed in frustrated ferromagnets.

### 4.1. Vortex motion in a superfluid/superconductor

Here we discuss this aspect of topological terms in the simple context of a vortex in motion within a superconducting or superfluid thin film. (Though we focus again on the 2D case, generalization to 3D is straightforward.) Recall from our previous discussions that the topological term for this problem reads (being interested here in the equation of motion, we are switching to the real time formalism)17
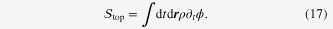
We assume for simplicity that the time dependence of the phase *ϕ* comes solely from the change in the vortex position 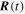, i.e. 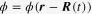. Noticing that 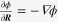, we can rewrite the topological term as18
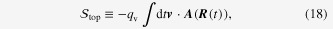
where 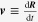 is the vortex velocity, 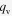 is the vorticity, and19
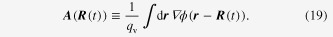
Equation (18) has precisely the same form as an action describing the coupling between a point charge of strength 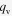 moving with a velocity 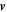, with a magnetic field which is represented by the vector potential 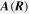. We therefore deduce [11, 15] that the vortex suffers the pseudo-Lorentz force20
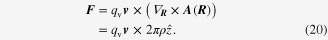
(An equivalent but more formal procedure, which will be used shortly, would be to resort to the principle of virtual work and calculate 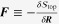.) This is in full agreement with the physical picture arising from the AB effect which the Berry phase factor of equation (8) describes; the fictitious magnetic field is very much real to the vortex. The pseudo-Lorentz force plays an important role in the dynamics of vortices. For example it has been argued that it influences the Hall conductivity of the cuprate high temperature superconductors in the mixed state [16], where the role played by the bound states at the vortex core must also be incorporated.

### 4.2. Motion of skyrmions in 2d

**Figure 6.**
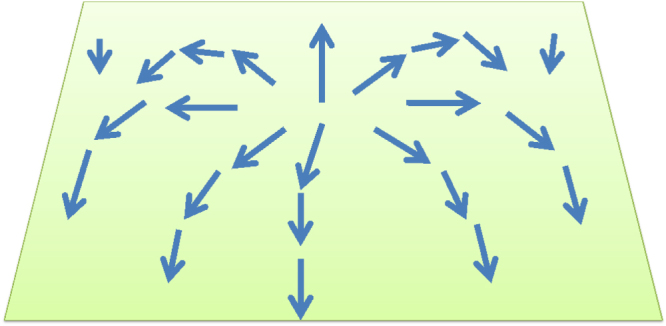
Schematic illustration of a skyrmion configuration in a ferromagnet. Skyrmions are spin configurations for which the winding number 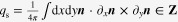, which counts the number of times the unit vector 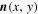 (indicated by the arrows) wraps around the unit sphere, is nonzero. In the configuration depicted, 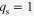.

A very similar force also arises in the dynamics of skyrmions in 2D ferromagnets [17], which are topological objects that are recently receiving considerable attention due to their potential value toward applications. Here the topological term takes the form (in real time) 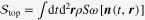, where *ρ* is the density of spins (whose spin quantum number is *S* and its direction is represented by the unit vector 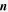) and 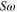 is the Berry phase term associated with a spin residing at position 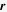. The skyrmion is a configuration for which the winding number associated with the snapshot configuration (i.e. instantaneous configuration)21
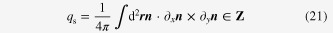
is a nonzero integer. (Notice that mathematically this is the same winding number (though associated with a different base manifold) which appeared in the previous section when we discussed the Haldane gap of antiferromagnetic spin chains.) A typical example of a skyrmion with 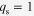 is depicted in figure 6. Since this integer-valued number cannot change continuously, a skyrmion is stable unless processes involving singular configurations (which usually are energetically too costly to be relevant) are allowed. As in the vortex dynamics problem, we assume that the time dependence of the field takes the form 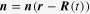, where 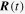 now stands for the center of the skyrmion (or more generally a collective coordinate of the skyrmion). Using the formula for the variation of the surface angle (see figure 5) 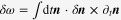, we readily find that22
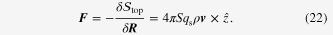
Thus a skyrmion in motion behaves like a charged particle in the presence of a magnetic field 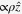.

The construction of equation (22) generalizes easily to 3D. Although the physical context where this situation may arise is as yet unclear, we outline its derivation, anticipating the future realization of a 3D analog of the skyrmion. This also serves as an example of how topological phenomena occurring in different dimensions are intimately related through their mathematical structures. (Readers uninterested in technical details can skip the remainder of this section.) We construct our 3D system (using cartesian coordinates 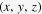) by imagining that for each 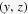, there is a 1D chain extending in the z-direction. Furthermore, we assume that each of these 1D chains accommodate an SU(2) Wess–Zumino–Witten model, which typically arises in studies of antiferromagnetic spin chains. The latter can equivalently be written as an O(4) nonlinear sigma model (i.e. a model featuring a unit-length vector field 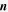 with four components) with the topological term23
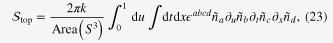
where 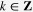, 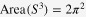 is the surface area of the unit three-sphere, and 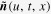 is an extension of the field 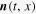 which satisfies24
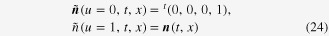
and interpolates smoothly as a function of the auxiliary variable *u* between these two limits. That this is a natural generalization of the spin Berry phase term becomes apparent when we rewrite the latter in an analogous manner, 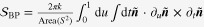, where 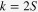 and 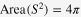. The way to extend the three-component unit-length vector 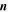 to 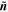 should be obvious if one follows the example of equation (24). The variation of equation (23) with respect to the change 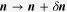 yields an expression similar to that for the spin Berry phase term, and is given by25
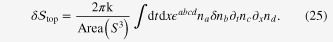
With this preparation we come back to the coupled-chains situation for which the topological term from each chain add up into the form26
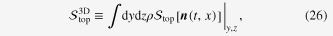
(*ρ* is the areal density (within the yz-plane) of these 1d extended objects), and consider a configuration in 3D (*xyz*) space for which the following winding number is a nonzero integer.27
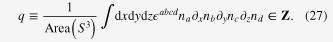
Introducing again a collective coordinate 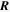 which represents the position of this solitonic object (a 3D generalization of the 2D skyrmion), we arrive once more at the now familiar form of force,28
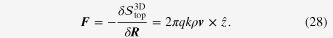



## 5. Surface states

Some of the topological terms that we encounter can be expressed as a total divergence. Under periodic boundary conditions this property often allows us to associate the term with a topological winding number; this was the case for the topological term (usually referred to as a *θ* term) which appeared in the Haldane gap problem. However, actual physical systems have a surface, in which case a total divergence term will contribute a surface action. The appearance of surface effects which reflect the topology of the bulk (the bulk–surface correspondence) is another characteristic feature of systems which are governed by topological terms.

### 5.1. Haldane gap

Let us revisit equation (13), the topological term for a spin *S* antiferromagnetic spin chain. We saw that under a periodic boundary condition, this leads to a *θ* term which discriminates between the behavior of integer and half-odd integer *S*. Let us now consider instead a spin chain with *open ends*. The topological term apparently reads, upon carrying out the spatial integration,29
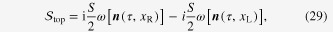
where 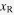 and 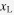 are the two edges of the chain. One recognizes that the two terms appearing in the right hand side are precisely the Berry phase terms for two spin moments, each residing at 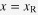 and 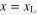, and carrying a spin quantum number of 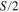. This suggests that fractional spin degrees of freedom are induced at the ends of a finite-length spin chain [18]. The emergence of 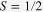 edge spins in *S* = 1 antiferromagnets has been verified experimentally as well as numerically. It should be noted that this simple argument is only valid for integer *S*, where there is a spectral gap and an associated finite correlation length (the bulk of the system is unaffected by the presence of the boundary). For half-odd integer *S*, with an infinite correlation length, the chain end effects are more subtle and require a detailed treatment.

### 5.2. Topological insulator

As there are by now a considerable number of accessible reviews on topological insulators [19], we will not go into their details, and merely remark that here also, several features similar to the edge state physics of Haldane gap systems appear. One example is the electromagnetic Hall response at the surface. The action governing the gauge response of a 3D topological insulator with a time reversal symmetry contains the unconventional term (which is also called a *θ* term, or an axion term depending on how one interprets the action)30
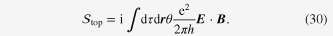
(Readers may wonder why this term becomes purely imaginary when turning to the imaginary time framework. This can be understood by recalling that *A*_0_, the time component of the electromagnetic gauge field (i.e. the scalar potential) should transform like 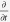, and thus 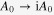 and accordingly 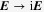 (while 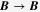) upon switching to Euclidean spacetime.) It is well known that this term, like the *θ* term of the spin chain problem, is a total divergence if *θ* is constant. More precisely, it is the divergence of the abelian Chern-Simons term which famously describes a quantum Hall response. From the requirement of time reversal symmetry, it can be shown that *θ* can only be (modulo 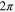) either 0 (conventional insulator) or *π* (topological insulator). This is analogous to the fact that the coefficient *θ* in the spin chain problem is also restricted to these two values due to spatial inversion symmetry (unless a bond alternating exchange interaction is introduced). Since the vacuum can be regarded as a conventional insulator, it follows that there is a jump in this value by the amount 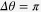 at the surface of a topological insulator. Thus there arises a surface Chern–Simons term which yields a half-integer quantized Hall effect 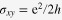.The observation of this effect requires that the surface state be gapped by a suitable perturbation, such as placing a magnetic thin film on the sample surface [19].

Finally we note that a topological field theory describing the physics of the matter field of topological insulators has also been proposed [20].

## 6. Topological structure of wavefunctions

In the previous section, we saw that when the topological term is a total divergence, it can induce an edge state at the spatial surface of the system. As was noted by Xu and Senthil [21], a similar surface contribution will arise at a temporal surface if the situation at hand requires that we have an open boundary in the (imaginary) time direction. Recall in this regard, that the path integral representation of a ground state wavefunction which we mentioned back in section 2 meets this condition: since we need to specify the initial and final boundary data of the field, the temporal boundary condition is by construction, not periodic.

The way in which temporal surface terms influence the ground state wavefunction (or wavefunctional) has interesting applications [21] to the study of *symmetry protected topological states* (SPT states) [22], which are the conceptual generalization of topological insulators (the basis of which rests on the framework of noninteracting electrons) to interacting systems. The concept of SPT states need not be restricted to electronic systems but can also apply as well, for instance, to bosonic and magnetic systems. As our final application of topological terms, we will briefly revisit the 1D version of the magnetization plateau problem of section 3.3 from this perspective, highlighting results from our recent work [23]. For details we refer the reader to the original reference.

We will hereafter concentrate exclusively on the magnetization plateau state, which according to our earlier discussion imposes on our system the condition 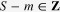. Let us also recall that basically, low-energy physics only involves spin fluctuation within the plane perpendicular to the applied magnetic field. This means that out of the total spin moment *S* residing at each site of our 1D system, a portion *m* is segregated off into a higher energy sector by the external field. It therefore follows that in the low-energy sector, we are left to deal with an effective subsystem consisting of a planar antiferromagnetic chain with spin quantum number 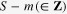 in the *absence* of a magnetic field (since the planar spin component is unaffected by the Zeeman coupling). With this substantial simplification in hand, we can make contact with the effective action for the Haldane gap system of section 3.4, where we are to make the following two modifications: (1) the spin quantum number *S* should be replaced by 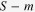, and (2) the planar limit must be taken. Carrying out the second modification properly is a subtle procedure which has been detailed elsewhere [10, 23], and we will go directly to the result for the imaginary-time effective action obtained by the strategy just outlined:31
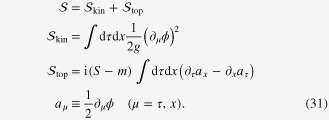
Notice that this action is a total divergence and will, in the presence of an open boundary, generate surface contributions. For the sake of simplicity we will take the strong coupling limit 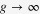, so that the action is dominated by the topological term. Using the prescriptions of section 2, we find that the ground state wavefunctional takes the form (we employ a periodic boundary condition in the spatial direction)32
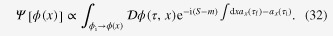
The action appearing in the exponent are the temporal surface terms mentioned at the outset of this section. Since the initial data is not relevant for our purpose, we need only concern ourselves with the final data, which results in33
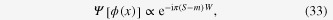
where 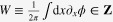 is a winding number recording the number of 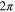 rotations that the angular field *ϕ* executes along the *x*-axis in the snapshot configuration 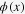. The dependence of equation (33) on *W* differs dramatically depending on whether 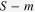 is odd (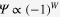) or even (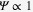). Since this structure is only dependent on the topological winding number *W*, it is expected to persist when perturbations of moderate strength are applied to the system. The fact that there are no ways to adiabatically connect these two wavefunctionals suggests that they describe different phases. (Furthermore, given that the topological term plays no role in determining the global structure of *Ψ* (i.e. the dependence on the topological sector labeled by *W*) for even 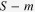, we expect that the ground state belongs to a topologically trivial phase, while the 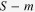 odd system is expected to lie in a nontrivial phase). This expectation is confirmed numerically as well as by a rigorous analysis of a solvable model [23].

Finally let us turn to the symmetry aspects of the present problem. Due to the presence of a magnetic field, time reversal symmetry is explicitly broken. Meanwhile the system does respect the symmetry with respect to an inversion about the center of a link connecting adjacent spins. To see how this symmetry affects the ground state, we break it by switching on a staggered magnetic field. This perturbation will induce a staggered magnetization 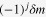 (where *j* is the site index) along the chain. By repeating the evaluation of the ground state wavefunctional for this case, we find that it now modifies to34
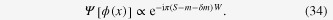
We thus find that by sweeping the value of 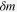 by tuning the staggered magnetic field, we can smoothly interpolate between the two types of wavefunctionals which was not possible in the absence of the staggered field (i.e. as long as the link-center inversion symmetry was respected). By employing a duality transformation technique, we can further show [10, 23] that it is possible to carry out this interpolation without encountering a gap-closing point (i.e. a quantum phase transition), which indicates that the two groundstates now belong to the same phase. We thus conclude that the odd 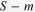 plateau is (1) a topologically nontrivial state belonging to a phase distinct from an even 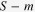 plateau state which is (2) protected by link-centered inversion symmetry. This is similar to the topological insulator story: the 3D 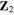 topological insulator, which belongs to a phase to be distinguished from a conventional insulator, owes its integrity and robustness to the time reversal symmetry. Once that symmetry is broken, the distinction between the topological and conventional insulators is lost.

We thus see that the recent development centering around topological insulators and superconductors provides us with new insights for exploring topological effects (especially on their robustness) in a broader range of condensed matter systems, including those which have been known for several decades. Most of the physical examples of SPT states identified so far are 1D systems. The identification of higher dimensional SPT states, along with their complete characterization and classification, is an important problem left for the future.
